# Dengue fever: new paradigms for a changing epidemiology

**DOI:** 10.1186/1742-7622-2-1

**Published:** 2005-03-02

**Authors:** Debarati Guha-Sapir, Barbara Schimmer

**Affiliations:** 1 Department of Public Health and Epidemiology Université catholique de Louvain 3094 Clos chapelle aux champs 1200 Brussels Belgium; 2 WHO Collaborating Centre for Research on Epidemiology of Disasters Dept of Public Health and Epidemiology Université catholique de Louvain 3094 Clos chapelle aux champs 1200 Brussels Belgium

## Abstract

Dengue is the most important arthropod-borne viral disease of public health significance. Compared with nine reporting countries in the 1950s, today the geographic distribution includes more than 100 countries worldwide. Many of these had not reported dengue for 20 or more years and several have no known history of the disease. The World Health Organization estimates that more than 2.5 billion people are at risk of dengue infection. First recognised in the 1950s, it has become a leading cause of child mortality in several Asian and South American countries.

This paper reviews the changing epidemiology of the disease, focusing on host and societal factors and drawing on national and regional journals as well as international publications. It does not include vaccine and vector issues. We have selected areas where the literature raises challenges to prevailing views and those that are key for improved service delivery in poor countries.

Shifts in modal age, rural spread, and social and biological determinants of race- and sex-related susceptibility have major implications for health services. Behavioural risk factors, individual determinants of outcome and leading indicators of severe illness are poorly understood, compromising effectiveness of control programmes. Early detection and case management practices were noted as a critical factor for survival. Inadequacy of sound statistical methods compromised conclusions on case fatality or disease-specific mortality rates, especially since the data were often based on hospitalised patients who actively sought care in tertiary centres.

Well-targeted operational research, such as population-based epidemiological studies with clear operational objectives, is urgently needed to make progress in control and prevention.

## Introduction

Dengue is the most important arthropod-borne viral disease of public health significance. Compared to nine reporting countries in the 1950s, today the geographic distribution includes more than 100 countries worldwide. Many of these had not reported dengue for 20 or more years and several have no known history of the disease. The World Health Organization (WHO) estimates that more than 2.5 billion people are at risk of dengue infection. Most will have asymptomatic infections. The disease manifestations range from an influenza-like disease known as dengue fever (DF) to a severe, sometimes fatal disease characterised by haemorrhage and shock, known as dengue hemorrhagic fever/dengue shock syndrome (DHF/DSS), which is on the increase. Dengue fever and dengue haemorrhagic fever/dengue shock syndrome are caused by the four viral serotypes transmitted from viraemic to susceptible humans mainly by bites of *Aedes aegypti *and *Aedes albopictus *mosquito species. Recovery from infection by one serotype provides lifelong immunity against that serotype but confers only partial and transient protection against subsequent infection by the other three. First recognised in the 1950s, it has become a leading cause of child mortality in several Asian and South American countries.

The average number of DF/DHF cases reported to WHO per year has risen from 908 between 1950 and 1959 to 514,139 between 1990 and 1999. The real figure is estimated to be closer to 50 million cases a year causing 24,000 deaths. Of an estimated 500,000 cases of DHF/DSS requiring hospitalisation each year, roughly 5% die according to WHO statistics. Regional distribution of dengue and its serotypes are described elsewhere [1,2]. In summary, DF/DHF/DSS is an immediate problem in south and southeast Asia and Central and South America. Although DF is present in the African region, there are no cases or outbreaks reported to WHO [3].

Half the world's population lives in countries endemic for dengue, underscoring the urgency to find solutions for dengue control. The consequence of simple DF is loss of workdays for communities dependent on wage labour. The consequence of severe illness is high mortality rates, since tertiary level care required for DHF/DSS management is beyond the reach of most of the persons at risk.

This paper reviews the changing epidemiology of the disease, focusing on host and societal factors and drawing on national and regional journals as well as international publications. It does not include vaccine and vector issues. Although each one of the issues taken up below merits an independent, in-depth treatment, we have selected only those issues where the literature raises challenges to prevailing views and therefore require further research, particularly given that most of these issues are key for improved service delivery in poor countries.

## Analysis

### Clinical presentation

Dengue infection can cause a spectrum of illness ranging from mild, undifferentiated fever to illness up to 7 days' duration with high fever, severe headache, retro-orbital pain, arthralgia and rash, but rarely causing death. Dengue Haemorrhagic Fever (DHF), a deadly complication, includes haemorrhagic tendencies, thrombocytopenia and plasma leakage. Dengue Shock Syndrome (DSS) includes all the above criteria plus circulatory failure, hypotension for age and low pulse pressure. DHF and DSS are potentially deadly but patients with early diagnosis and appropriate therapy can recover with no sequelae. Case management for DF is symptomatic and supportive. DHF requires continuous monitoring of vital signs and urine output. DSS is a medical emergency that requires intensive care unit hospitalisation [4].

The increase in dengue mortality is considered to be a reflection of the increase in the proportion of DF patients who develop DHF/DSS. The pathogenesis of DHF/DSS is widely considered to be antibody-dependent enhancement in secondary infection with a virus of different serotype [5]. Evidence in support of this comes from many studies including from the Cuban epidemics of 1981 and 1997 [5,6] and a five-year study of Yangon (Myanmar) [7]. However, absence of a significant association between secondary infection or co-circulation of different serotypes and DHF/DSS has also been noted [8,9]. The disease is widely considered to be associated with secondary infection and co-circulation of several serotypes.

Alternative or additional factors associated with severe illness, such as high viraemia titres, have also been suggested [10]. So far, this has been associated with secondary infection as demonstrated by Vaughn *et al*., and Libraty *et al*. [11,12]. On the other hand, one expression of higher viral virulence could be higher viraemia leading to greater severity, but this has not not yet been demonstrated (Guzmán, 2003 personal communication).

Viral virulence [13], immunological responses and increased pathogenicity of specific serotypes [14] have been implicated as critical for the appearance of DHF. This has been found for the three serotypes DEN1, [13] DEN 2 [15] and DEN 3 [8,10,16,17], but so far not for DEN 4 [18].

The evidence from different studies also shows that the pathogenesis of DHF/DSS may be multi-factorial and understanding remains incomplete.

### Epidemiological changes

Demographic, economic, behavioural and social factors are often keys for effective communicable disease control and underpin successful public health programmes. Despite promising indications in the literature, these factors have remained poorly understood in the case of dengue. Furthermore, recent field evidence raises some questions regarding widely accepted characteristics of dengue that need review and confirmation.

#### Shift in modal age

DF is typically acknowledged to be a childhood disease and is an important cause of paediatric hospitalisation in southeast Asia. There is, however, evidence of increasing incidence of DHF among older age groups. Since the early 1980s, several studies in both Latin America and southeast Asia have reported a higher association of DHF with older ages. The earliest studies were by Guzmán (1981) in Cuba and Rigau-Pérez in Puerto Rico [6,19]. Later on similar observations were noted in Nicaragua and Brazil. In some southeast Asian countries where dengue has been epidemic for several years, this age shift is clearly observed, indicating an epidemiological change in dengue infection in those locations [20-22].

Three studies in Asia using surveillance data report increasing age of infected patients. In Singapore, surveillance data showed a shift in peak dengue mortality from paediatric ages (1973–1977) to adults in 1982, since which year more than 50% of the deaths occurred in patients older than 15 years. From 1990–96, the highest age-specific morbidity rates were in the 15 to 34 year age groups [23]. In Indonesia, surveillance data from 1975 to 1984 showed an increase in incidence rates among young adults in Jakarta as well as in the provincial areas [24]. Adults have accounted for proportions as high 82% of all cases in the hospital-based surveillance study during the 2000 epidemic of dengue in Bangladesh [25]; the highest proportion of cases occurred in the 18 to 33 year age group. All deaths in the Bangladesh outbreak in 2000 were in persons older than 5 years. In Puerto Rico, surveillance data analysis showed the highest incidence rate (11.8/1000) in the 10–19 year age group during an outbreak in 1994 and 1995 [26].

Hospital-based studies have similarly reported increasing infection rates among adults, mentioning that it is contrary to the popular belief that dengue is a paediatric disease [27,28]. The trend for increased incidence among young adults has important implications for control and prevention. Whether these are real increases (based on population distributions), increases in the proportion of DHF/DSS (and, hence, the proportion hospitalised) but not DF, or the result of improved classification and diagnosis needs clarification. Comparative incidence and case fatality ratios (CFRs) of severe illness in adults and children and the economic implications are discussed later.

#### Racial predisposition

Race-related susceptibility to dengue has been observed in a few studies and merits further investigation. In a retrospective seroepidemiologic study Guzmán reported that blacks and whites were equally infected with DEN-1 and DEN-2 viruses during the Cuban epidemics of 1977 and 1981, while severe dengue disease was observed less frequently in dengue-infected black persons than whites [5,6].

A study in Haiti observed that despite virologic pre-conditions hypothesised to be precursors for DHF (i.e. the evidence of previous infection by DEN virus types 1, 2 and 4), local children did not develop severe illness [29]. The authors concluded that this finding provides further evidence of a dengue-resistant genotype in black populations. In 1998 the Los Angeles County vital registration system reported DF/DHF incidence, but only among Hispanic and white ethnic groups (0.1 and 0.07/100,000) [30].

Genetic polymorphism in cytokine profiles and coagulation proteins has been proposed as a factor protecting persons of African origin [31]. Evidence for this hypothesis has been found in meningococcal disease, in which a genetic polymorphism in the gene encoding an essential protein involved in coagulation is a predictor for developing severe disease with lethal outcome.

In Asia, two studies report racial differences in disease incidence. A 15-year study of the epidemiology of dengue reports a significantly higher incidence of DHF among Chinese compared to Malaysian males [32]. This finding was supported by a six-year surveillance data study in Singapore, which found the race-specific morbidity rate among the Chinese to be three times that of the Malays and 1.7 times that of Indians [23]. Although none of the above constitutes convincing evidence for the hypotheses, they highlight a useful area for better understanding of dengue pathogenesis and health service planning.

#### Sex differences

Understanding male-female differences in infection rates and severity of disease is important for public health control programmes. A few hospital-based studies and surveillance data show a male-female difference in infection rates and in severity of disease. Three independent studies from epidemics in India and Singapore found nearly twice the number of male patients compared to females (Lucknow and Singapore both report male to female ratios of 1.9:1 and Delhi 1:0.57) [33-35]. In his hospital-based study during the 1996 epidemic in Delhi, Wali reported an even higher ratio of 2.5:1 [27]. Another study during the same epidemic found a male to female ratio of 1:0.25 cases for DSS. However, of the three deaths in this sample, two were female [35]. Surveillance data from Malaysia revealed a male preponderance among Indian and Malay patients (1.5:1), but the ratio was almost equal for those of Chinese origin [32]. The Ministry of Health, Bangladesh reported a hospital patient DF/DHF male to female ratio of 1.5:1 during an outbreak in Chittagong in 1997 [36], although a later study of DHF only during the 2000 outbreak found no differences between sexes [37]. With the exception of the study by Shekhar, all the others were hospital-based and may represent those who sought care rather than the infected population [27].

Studies in South America generally report that both sexes are equally affected [26,38] although a male to female ratio of 0.65:1 was described as "typical" for dengue [9]. Kaplan, in a rare study testing for significance, found a higher proportion of women in all of his four Mexican samples (p < 0.001) [39].

Of significance are two studies in Asia by Kabra and Shekhar where severe illness and CFR were consistently higher among females despite higher incidence in males [32,40]. Halstead [41] had pointed out as early as 1970 that males predominate among those with milder disease but females account for more severe illness. He suggested that either immune responses in females are more competent than in males, resulting in greater production of cytokines, or the capillary bed of females is prone to increased permeability. Kaplan in Mexico suggests that an incidence bias in favour of females is related to the timing of the survey interviews, while Goh puts forward that low incidence among women occurs because they stay at home and are less exposed to infection [39,42].

No studies suggest gender bias in home care and male preferences in health care seeking, still prevalent in many Asian and other traditional societies. It is widely recognised that in many of the Asian communities, lower disease incidence in women may be a statistical artefact related to lower reporting and care-seeking for women from traditional practitioners who do not report to public surveillance systems. By the same token, women are less likely to be taken for care at a hospital when ill or are taken at late stages of disease, when no other options are available. Determining sex differences, both in infection and severity of disease, requires well-designed and targeted studies to capture both biological and social factors that drive disease patterns in a community.

#### Rural spread

Historically, DF/DHF has been reported as occurring predominantly among urban populations where density of dwellings and short flying distance of the vector create the right conditions for transmission. However, the literature shows that dengue transmission and, in some cases, outbreaks occur in rural settings in both Asia and Latin America. In the WHO Western Pacific region, WHO has confirmed that disease spread into rural areas from where it had not been reported previously [20].

Rural epidemics occurred as early as 1976 in Indonesia, and in 1994 the outbreak in Laos began in a remote, rural district of Nasaithong [43,44]. Today, Thailand has an incidence rate that is higher in rural (102.2 per 100,000) than urban areas (95.4 per 100,000) [28]. Similarly, in India, entomological investigation showed a widespread distribution of *Aedes aegypti*, both in rural and urban areas during an outbreak in Gujarat in 1988 and 1989 [45]. Increase in DF/DHF among rural populations is also observed in Central and South America and identical rates in both populations are reported [9]. Among jungle dwellers in Peru, antibody prevalence up to 67% compared to 66% among the urban population have been found [46].

In industrialised settings, the Centers for Disease Control and Prevention (CDC) reported an outbreak of DF among residents of the rural towns of Hana and Nahiku in Hawaii in 2001. The outbreak was historically unusual because infection occurred among residents who have no history of recent travel and the *Aedes aegypti *mosquito has not been seen in Hawaii since it was supposedly eradicated by pesticide spraying in 1943 [47].

Increased transport contact, mobility and spread of peri-urbanisation have been the most frequently cited reasons for spread of dengue to rural areas [48]. While some rural incidence linked to travel contact with urban areas is conceivable, outbreaks and infection rates equal to those in urban areas warrant further investigation. Improved reporting could also be a factor, but it would be less likely in areas such as Hawaii, USA. Standard epidemiological techniques such as spatial studies of cases and careful patient histories could shed further light into transmission patterns in rural populations. Health service structures and utilisation patterns differ substantially between urban and rural areas in many tropical countries and contextually appropriate strategies will be required for effective impact.

#### Seasonality and climate variability

The incidence and, in particular, epidemics of dengue have been commonly associated with the rainy season, and the El Niño phenomenon has been incriminated in the increases of certain vector-borne diseases, including dengue [49,50].

Despite the number of studies, convincing data or models supporting these hypotheses are scarce. The relationship between temperature, rainfall and vector-borne disease are increasingly seen as oversimplifications. A study modelling DF transmission and seasonal temperature on data from Puerto Rico from 1988 to 1992 revealed weak relationships between monthly mean temperature and incidence of DF [51]. The study concluded that factors related to history of herd immunity, introduction of new serotype or demographic transitions influence transmission.

More recently, long-term meteorological trends were studied in four high-altitude sites in East Africa, where increases in malaria have been reported in the past two decades [52]. They did not observe any significant change in temperature, rainfall, vapour pressure and the number of months suitable for *P. falciparum *transmission in the past century or during the period of reported malaria resurgence. Others have questioned models linking global temperatures and disease incidence, stating that, historically, climate has rarely been the principal determinant of vector-borne disease prevalence. Neither does the literature provide an adequate evidence base establishing the impact of climate change on vector-borne disease [53,54]. The "bandwagon" of El Niño [55] and dengue incidence is now placed under scrutiny and further research will have to be done before climate variations can be nailed down as a culprit.

### Health systems issues

#### Socio-economic context

Social and economic factors play an essential role in the incidence and prevalence of DF and DHF. Air conditioning, screens and safe water supplies in wealthier countries help prevention and better health services reduce or eliminate mortality from DHF. Unplanned urbanisation and inadequate resources for vector control are factors that promote transmission and are characteristic of poor rather than richer countries. Reiter *et al*. (2003) studied dengue transmission on the Mexico-USA border and found higher rates in the Mexican city compared to the American one [56].

However, some anomalies persist despite the rich/poor divide in disease incidence. Despite energetic control programs in the wealthier endemic countries of southeast Asia such as Singapore, Malaysia and parts of China (eg. Hong Kong), dengue continues to be a problem. Malaysia reports some of the highest numbers of cases during epidemics compared to other countries in the region. In some of these cases, particular traditional practices, such as rainwater storage on roofs, expose them to higher risk.

The major epidemic in Puerto Rico in 1977 serves as a reminder that advanced public health capacities and economic development may not guarantee protection against massive epidemics [9]. Despite high quality of health services and richer circumstances, complacency in endemic countries may lead to increased rates without continued vigilance.

On an individual level, evidence points to greater susceptibility among well-nourished or middle-class communities rather than malnourished and poorer patients commonly associated with other tropical diseases.

A case-control study of serologically-confirmed DHF patients, other infectious diseases patients and healthy children in the Children's Hospital in Bangkok showed that malnutrition amongst DHF patients was significantly lower [57]. In India, a hospital-based study found no association between nutritional status and severity of illness [40].

Middle classes have been specifically noted as the proportionally predominant group during the epidemic in Dhaka Bangladesh [25] and upper social classes had statistically higher sero-infection rates in Fortaleza and San Luis epidemics in Brazil [38]. Confounding factors for the preponderance of DF/DHF among the upper classes or well-nourished dengue patients were not discussed in any of these studies.

Few studies specifically measure and test socio-economic determinants of exposure at community levels. Heukelbach in Fortaleza, Brazil did examine socio-economic variables but their study did not show an association with DF [58]. Since all 34 cases selected were from a shanty town (favela), a lack of heterogeneity may have been a factor for this result rather than a real absence of difference. In Taiwan, Ko, also in a case-control study, observed that patients who lived near markets and/or open sewers or ditches had a risk of contracting disease 1.8 times higher than those who did not [59]. Since housing near sewers and ditches is likely to comprise poorer families, the analysis should have tested for house site while controlling for use of screens, which were significantly associated with incidence.

#### Costs

On a macro level, the impact of dengue on the economy is likely to be increasingly similar to that of malaria. Prevalent in communities characterised by subsistence or daily wage labour, a week's illness can be catastrophic for poor families. As a primarily paediatric disease in the past, the active labour force or the family wage earners were less affected. Now, as the modal age of illness and incidence increases, losses in productivity and earning capacity may be expected. The economic lesson from malaria was learnt late and when the resurgence was already in full swing. Dengue fever risks the same fate.

With regard to costs of care, few economic studies exist and most estimate economic loss to range in millions. Von Allmen *et al*. undertook a cost analysis of the epidemic of DF/DHF in Puerto Rico in 1977 using upper and lower limits of incidence [60]. They estimated the direct costs (medical care and epidemic control measures) to range between US$2.4 and $4.7 million and indirect costs (lost production of patients and parents of children) between US$6 and $15 million. Another economic study, still in Puerto Rico, assessed the loss in terms of DALYs due to dengue [61]. At 658 DALYs per year per million population, the study concluded that, in terms of its magnitude, DF ranks with TB, STDs (including HIV), childhood diseases or malaria.

On a micro level, a detailed study on costs of care in 3 hospitals in Bangkok estimated direct adult patient costs at US$67. Including opportunity costs, this figure increased to US$161.49. The net hospital cost for each DHF patient was US$54.60 and the public sector cost of prevention and control of the outbreak was US$4.87 million. The total expenditure for DHF in 1994 was estimated to be at least US$12.56 million, of which 45% was borne by the patients [62].

These figures are reminders that most of the countries subject to DF/DHF cannot realistically afford a US$5 million prevention and control budget for a single disease and that the monthly income of many families in these countries is less than the direct cost of US$70 a month.

#### Knowledge, attitude and practice (KAP)

Much needs to be done in finding effective strategies for behaviour change. Since mothers are the first-line care-givers, this aspect is key, particularly for childhood diseases. KAP studies are rare and therefore little is known regarding knowledge and attitude of the exposed population towards dengue. However, the little that is known is encouraging.

Straightforward community education to reduce breeding sites for mosquitoes performed better than chemical spraying in a controlled experiment in Mexico [63]. However, housewives, the unemployed and the elderly had significantly lower levels of knowledge of the disease compared to students and persons of younger ages (odds ratio (OR) = 0.44, 95% confidence interval (CI): 0.31–0.64). Other KAP studies have found that radio and television are very effective channels for knowledge dissemination. Nevertheless, these same studies found that while communities can score well in knowledge of the disease, they perform less well in attitude and practice, indicating that behaviour change is one area to target in social mobilisation programmes [64-67].

Treatment-seeking behaviour and lay symptom assessment is the first step in the chain to early diagnosis and was found to have an impact on duration of illness in Thailand [68]. In that context, it is discouraging to note that 45% of individuals in a population-based survey (23,970 households) in the urban municipality of Vientiane did not know what action to take when their children are diagnosed with dengue or what they should do for prevention [44].

Finally, reducing mortality from DHF and strengthening its control and prevention clearly cannot be done by the population alone. In most circumstances, these are poor populations with other pressing agendas. The programme requires public sector leadership with strong intersectoral collaboration. The WHO has made important progress to determine ways and mechanisms through which to achieve collaboration between sectors and state policy directions for control.

#### Trends in case fatality rates

Two aspects present themselves for useful discussion in this area. One relates to wide variations in CFRs between countries, sub-national units and hospitals under similar virological conditions. The other relates to differential risks of severe illness and mortality between children and adults.

The global case-fatality rate (CFR) for DHF/DSS has been declining in most of the endemic countries according to government statistics. The overall CFR in the southeast Asia region is now less than 1% [20]. However, disaggregated data reveal a different picture. Rates vary significantly between countries, provinces and hospitals, pointing to a more complex situation.

From 1995–2000, the CFR in the countries of WHO Western Pacific Region ranged from 0.06% in Singapore and 0.17% in Malaysia to 3.4% in Cambodia. Hong Kong reported no deaths [69]. In Vietnam, province-based 1998 data for DHF show CFR ranging from nearly 13% in Ha Tinh to 0.5% in Quang Tri [70]. Although the four provinces with the highest CFR were at some distance from Ho Chi Minh City or Hanoi, the four of the lowest were not particularly closer to these centres of tertiary care. In Laos, on the other hand, CFR for DHF during 1998 reached a high of 9.7% in Champassak province compared to 1.4% in Municipality of the capital city, Vientiane [44]. Wide variation in CFRs ranging from 0.1% to 5%, was also noted between the first administrative divisions in the Philippines [71].

During the 1998 epidemic in Cambodia the CFR in Kantha Bopha, a private, charitable hospital, was substantially lower (1.96%) than the national average (2.91%) [70]. Inter-district and inter-hospital variation is generally indicative of quality of care. Availability of medical supplies, equipment and economic status of patients can explain some differences but analyses to distinguish between the performances of provinces and countries in comparable settings would be useful for designing more effective disease control.

Secondly, studies have postulated higher risk of DF/DHF morbidity and mortality among children compared to adults [15]. Recently, increasing reports of severe illness among adults and in some cases higher CFRs (e.g. age-specific CFRs from San Lazaro hospital over one year were 3.8% for 35–39 year olds, 8% for over 45s compared with 2% and 2.6% for 0–4 and 5–9 year age groups) merit closer looks at determinants of adult mortality [37,71,72].

#### Case management and early detection

In addition to vector control, widely recognised as a preventive strategy of choice, key health sector response for reduction of mortality and morbidity lies primarily in two areas: early detection (including care-seeking behaviour change and better surveillance) and improved case management of patients. Mortality in excess of 1% may be considered the consequence of inadequate care, late diagnosis and delayed hospitalisation.

A hospital-based study during the dengue outbreak in Delhi revealed that mortality could be very low in patients who came early to the hospital [73]. Late presentation was also strongly associated with increased mortality in children with DHF in the Philippines [74].

The short interval between onset of haemorrhage and death, especially in young children, makes rapid medical intervention for DHF/DSS a critical factor for survival. For most communities at highest risk of disease, intensive care facilities are only available at distant capitals requiring motorised transport, usually beyond the reach of many. Early diagnosis and leading indicators for DHF/DSS can ensure the availability of travel time to transfer the patient for effective treatment. Case-control studies have shown that low-normal hematocrit count at time of shock is a significant risk factor for haemorrhage [75] and potential predictors for clinical outcome, such as decrease in total plasma cholesterol, and high- and low-density lipoprotein, were associated with the severest cases [76].

However, research into predictive factors for severe illness is neither abundant nor conclusive. Moreover, as Van Gorp concludes, low capacity and lack of resources at secondary levels of health services limit the operational use of many of these findings [76].

At this time, the WHO classification of dengue diseases is often not feasible in many countries because of lack of trained health professionals, inadequate laboratories, and radiological support. Neither are facilities to detect DHF by using hematocrit and plasma leakage signs readily available in many tropical countries. As successful treatment of dengue depends on symptom recognition and careful fluid management, a simpler dengue disease classification scheme, realistic in poor, provincial conditions and better training of district-level personnel is needed.

A few creative approaches to primary health care to improve quality of care and case management at primary health care levels have been reported in the literature. For example, encouraging results have been found in Vietnam where they reduced dengue mortality rates by 64% through innovative primary healthcare concepts, including paediatric priority training units for medical staff, health education for patient carers and promotion of outpatient treatment to avoid unnecessary admissions [77]. Reduction of CFRs from 10–15% (40% in some areas) in the early 1950s to less than 0.5% today in east Asian referral hospitals have been attributed to better training of the hospital staff [78].

The effect of strengthened health systems is recognised by public health authorities including WHO but is missing operational research and policies to put them into effect.

#### Surveillance and reporting

Unreliable statistics are an extremely serious weakness from many perspectives. Estimates of DHF/DSS CFR from surveillance data are consistently lower than those from single sample study data suggesting under-reporting or misclassification of deaths. Inadequate knowledge of case definitions among district health personnel compromise complete reporting even within the public health service system. Inappropriate denominators further add to the confusion in estimating prevalence and incidences.

Reporting deviations can lead to seriously misleading CFRs in countries where reliable estimates are urgently needed for effective resource programming. In Laos, for instance, 8197 DHF cases and 24 deaths were registered by the WHO in 1996, compared to 2563 cases and 23 deaths registered by the Institute of Malariology, Parasitology and Entomology (IMPE) for a CFR of that is 3 times higher than WHO statistics [22], Most national surveillance data rely only on public sector institution reporting.

An evaluation of the dengue reporting system in Bandung, Indonesia (covering private and public hospitals) found that only 31% of hospitalised DHF/DSS cases were reported to the Municipal Health Authorities [79]. In Puerto Rico, a hospital record review revealed a ratio of 3:1 total DHF cases compared to those detected by surveillance [80]. More alarmingly, in Texas, USA, an assessment of underdiagnosis of dengue was undertaken motivated by an outbreak in a town across the border in Mexico. A review of medical records between 23 July and 20 August 1999 found that 50% of suspected cases had undiagnosed dengue infection. [81].

Based on the above studies, a conservative estimate would be that a third of the total cases are captured by surveillance systems, indicating that the global incidence rate could be around 1.5 million cases of DHF on an average year rather than the 0.5 million estimated by WHO.

While complete surveillance data may be an unrealistic option in many the affected countries, sentinel surveillance and sample surveys using reliable methodologies could be undertaken to provide more accurate estimates of the disease burden and fill in the gaps. Occasional sample surveys of the private sector could help better estimate the bias in disease burden.

## Conclusion

On 18 May 2002, the WHO General Assembly confirmed dengue fever as a matter of international public health priority through a resolution to strengthen dengue control and research.

Today, changing characteristics of the disease deserve serious research attention. Shifts in modal age, rural spread, social and biological determinants of race- and sex-related susceptibility have major implications for health service planning and control strategies. Behavioural risk factors, individual determinants of outcome and leading indicators of severe illness are poorly understood, compromising the effectiveness of control programmes. Early detection and case management practices have been noted as a critical factor for survival. Yet well-targeted operational research in these areas is rare. Population-based epidemiological studies with clear operational objectives should be launched as concerted efforts at regional levels.

A major weakness is the inadequacy of sound statistical methods in some of the reviewed studies. Samples are exceedingly small in many cases, selection methods are often inadequately described or are self selecting, tests of significance are frequently not undertaken or not reported and denominators are not clearly described. Conclusions therefore do not have the full benefit of objective statistical analyses, reducing the scientific strength of the results. Furthermore, conclusions regarding case fatality or disease-specific mortality rates are hard to draw since they are frequently based on hospitalised patients who had actively sought care in tertiary centres. However, a systematic approach and a clear international research agenda can quickly bring forward the frontiers of knowledge. Better understanding of the above will not only feed into operational policy for dengue control, but also provide fertile terrain for vaccine application strategies in the future.

Today, dengue control and prevention requires thinking outside the tropical disease box. Many of the affected countries are some of the poorest. Approaches that are realistic for their infrastructure need to be urgently developed.

## Competing interests

The author(s) declare that they have no competing interests.

## Authors' contributions

Debarati Guha-Sapir set out the plan of the paper, its focus areas and main messages. Barbara Schimmer carried out the literature search, summarised the studies and their results. She helped D. Guha-Sapir with the writing of the text.
